# Mr. Orsamus G. Warren

**Published:** 1892-06

**Authors:** 


Mr. Orsamus G. Warren, senior proprietor of the *Buffalo Commercial*, died at his residence in this city Friday, May 6, 1892, of pneumonia, aged forty-six years. Mr. Warren had been connected with the press since boyhood, and grew up under the masterful tutelage of his father, the late James D. Warren, whose name and fame were known throughout the land. Orsamus G. was his eldest son, and assumed the proprietorship of the *Commercial* at the death of his father, whose partnership he had enjoyed for many years. Mr. Warren was a strong personality, enumerating his friends by hundreds and thousands, and will be sadly missed in political, business, and newspaper circles.

Appropriate action, expressing sympathy for his family and appreciation of his worth, has been taken by the Merchants' Exchange, Buffalo Press Club, Republican League, American Exchange Bank, and several other business and social interests with which Mr. Warren was identified; while telegrams of condolence and editorials of sympathy have poured in from all quarters. Seldom has a citizen of Buffalo received such an avalanche of memorial tributes, and his sudden taking off will continue to be regretted by thousands of his fellow-citizens.

